# Volume Fourteen

**Published:** 1874-08

**Authors:** 


### Volume Fourteen.

With this number we enter upon the fourteenth volume of the BUFFALO MEDICAL AND SURGICAL JOURNAL. It has been the aim of the JOURNAL to furnish its readers with the best information upon the leading topics of the day. The new ideas constantly advanced in Medicine or Surgery, have been commended or condemned as they have proved valuable or worthless. With an eye to the prosperity and honor of the medical profession, it has jealously watched every avenue by which evil could enter the ranks, and has not hesitated to utter its warning when it has seemed necessary. The active observers in the professional ranks who have supported the JOURNAL by original contributions to medical literature, have our sincere thanks, and our earnest request for continued favors. We need not point out to our contributors the fact which must be evident to them, that in observing the course of disease or the action of remedies in order to make known the results they have not only benefited their professional brethren by their labors, but have enlarged, and strengthened their own capacity for observation.

What the success of the JOURNAL may be in the future, the future can only reveal. He who aims high even if he does not hit the mark attains a more exalted position than he who strives for a less ambitious end. Our measure of success lies in the amount of earnest, cordial support we received from the profession; what we shall attain we cannot foretell; judging by the past, the prospect is good, we shall then take the position that the wisest course lies in hoping and striving for the best, not in dreading defeat or failure. B.

